# Persistent Small Bowel Obstruction due to Small Bowel Adenocarcinoma: A Case Report

**DOI:** 10.7759/cureus.20233

**Published:** 2021-12-07

**Authors:** Orlando Fleites, Stephanie S Pelenyi, Charles K Lee, Christopher A Wisnik, Ammarah Tariq, Ameen Abdel-Khalek, Frederick M Tiesenga

**Affiliations:** 1 Medicine, St. James School of Medicine, Park Ridge, USA; 2 Surgery, West Suburban Medical Center, Oak Park, USA; 3 Anesthesia, Avalon School of Medicine, Willemstad, CUW; 4 Medicine, Saint James School of Medicine, Park Ridge, USA; 5 Medicine, Poznan University of Medical Sciences, Poznan, POL; 6 General Surgery, West Suburban Medical Center, Chicago, USA

**Keywords:** diverticulitis, bowel adhesion, colon cancer, ischemic bowel disease, small bowel obstruction

## Abstract

Small bowel obstruction (SBO), of both partial and complete types, is a condition predominantly caused by intra-abdominal adhesions and hernias. However, a known but very uncommon cause of SBO is malignancies, which are more complicated than those caused by adhesions and hernias, and associated with poorer prognoses; of these, small bowel adenocarcinoma is an even rarer etiology of SBO. The majority of SBO cases that are treated have resolution of symptoms and do not have recurrence/persistence of the condition; however, reports suggest that approximately one-fifth of SBO cases that are treated will result in recurrence/persistence of SBO requiring repeat admission.

Here we report the case of an 89-year-old female with a past medical history of right lower extremity deep venous thrombosis, inferior vena cava filter placement, iron deficiency anemia, diverticular disease, internal hemorrhoids, sick sinus syndrome, emphysema, hypertension, dyslipidemia, and hypothyroidism, who presented with diarrhea and intermittent dark stool. Abdominal computed tomography (CT) while in the emergency department initially showed possible ischemic bowel and SBO. After an exploratory laparotomy with small bowel resection and adhesiolysis, pathological analysis of a resected specimen showed infiltrating small bowel adenocarcinoma. Persistence of symptoms necessitated subsequent abdominal imaging, which demonstrated persistent SBO, which was treated with a second exploratory laparotomy with small bowel resection and end ileostomy.

## Introduction

SBO is a condition that is most commonly caused by intra-abdominal adhesions in the United States (globally, hernias are reported as the most common cause) [1]. Patients will often present with nausea, vomiting, abdominal scarring (from prior surgical procedures), hernia bulges, abdominal pain, a lack of bowel movements/flatulence, and abdominal distension [1-3]. SBO accounts for 4% of all surgeries due to abdominal pain per year in the United States; the most common etiologies of SBO include adhesions from prior abdominal surgery (70%), malignancy (10-20%), hernia (10%), and inflammatory bowel diseases (5%) [4]. The malignancies most frequently associated with bowel obstruction are those of the ovary, colon, stomach, pancreas, bladder, and endometrium [5]; in patients with previously treated colorectal cancer, approximately 10% develop SBO, of which half are estimated to be attributable to cancer recurrence [6].

The symptoms of SBO can be non-specific; therefore, additional diagnostic testing may be required to differentiate SBO from ileus or other differential diagnoses; if left untreated, complications of SBO can lead to strangulation and/or perforation of the small bowel [1-3]. SBO may be classified as partial or complete, and based on this, management will vary [1,3]. Partial SBO may be managed conservatively, including bowel rest and nasogastric tube (NGT) suction [1,4]. Complete SBO often requires surgery (roughly 50%-75% of cases) due to the high risk of strangulation and small bowel infarction [4]. SBO due to malignancy is an atypical entity, in that it often presents later than most other cases of SBO and may also have its etiology misattributed; estimates suggest that 22-33% of malignancy-induced SBO cases are diagnosed with adhesions as the etiology [7].

We present a case in which an 89-year-old female with an extensive past medical history including deep venous thrombosis (DVT), iron deficiency anemia, diverticular disease, sick sinus syndrome, emphysema, and hypothyroidism presented with diarrhea and intermittent dark stool. Initial imaging indicated possibly ischemic bowel due to SBO, and the patient underwent a successful exploratory laparotomy with small bowel resection and adhesiolysis. Pathological analysis of the small bowel specimen taken from this operation revealed grade 2 infiltrating adenocarcinoma developed on a tubulovillous adenoma, infiltrating and focally penetrating the muscularis propria. Persistent symptoms during the patient's postoperative course necessitated further imaging, which confirmed the persistence of SBO. The patient subsequently underwent another exploratory laparotomy with small bowel resection, this time with end ileostomy, which was successful.

## Case presentation

Chief complaint

Our patient is an 89-year-old female who was admitted to the emergency department due to multiple episodes of dark-colored diarrhea, which had been ongoing for two days.

History of present illness

The patient reported diarrhea following every attempted meal with onset two days prior to admission. The patient has no sick contacts or history of recent travel. The patient also reports that her stools are dark in color and denies abdominal pain. The patient is afebrile and reports no nausea, vomiting, or chest pain.

Past medical history

The patient’s past medical history was significant for hypothyroidism, dyslipidemia, hypertension, diverticulosis with diverticular bleeds requiring blood transfusion, sick sinus syndrome following pacemaker installation, DVT following inferior vena cava filter installation, iron deficiency anemia, and internal hemorrhoids. The patient also reported a history of cancer but was unable to elaborate on any further details of this condition, and no records relating to this condition were available.

Physical examination

On examination, the patient’s vital signs were within normal limits, and the patient was not in any acute distress. The patient was alert and oriented, with a soft and non-distended abdomen with no evident scarring or apparent hernias. Bowel sounds were normal. Of note was the placement of an NGT, which had been installed while the patient was in the emergency department, which had 50 mL of output in the 12 hours prior to the examination.

Investigations

**Figure 1:**
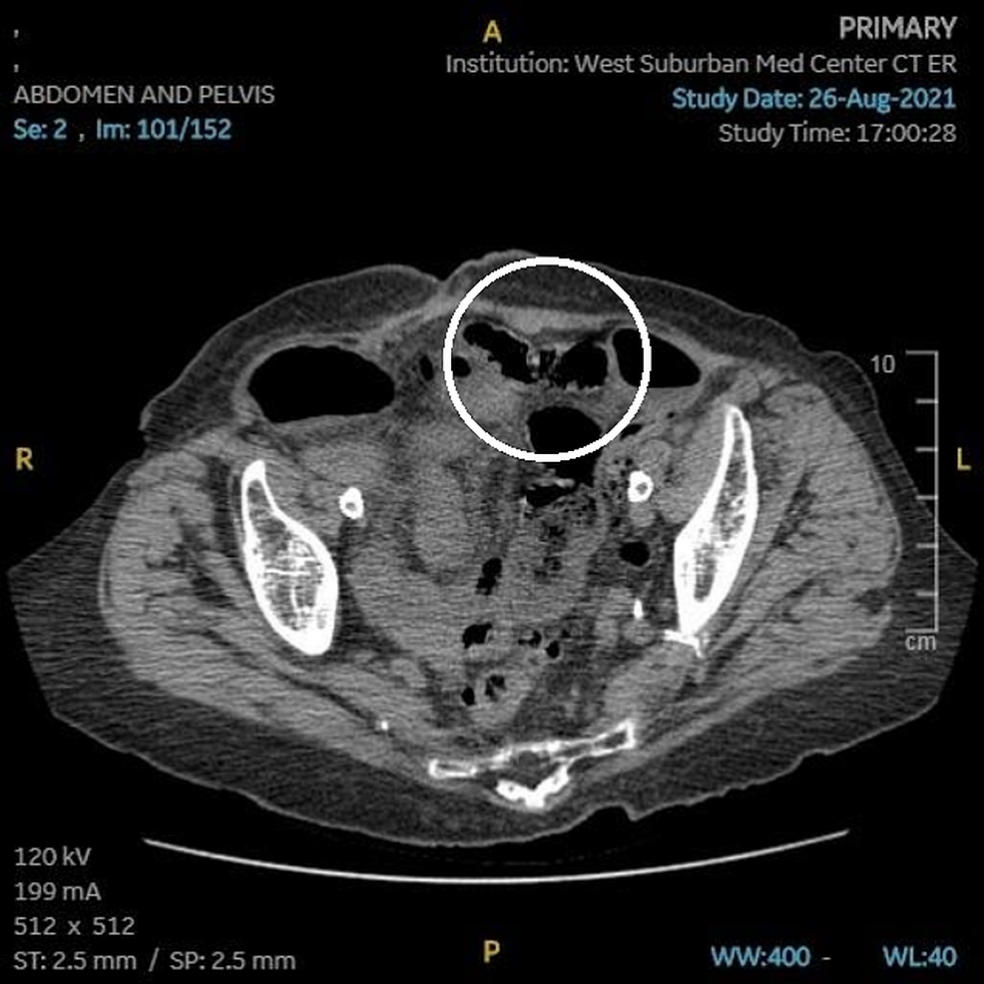
Computed tomography (CT) obtained prior to the first exploratory laparotomy, showing a segment of abnormal-appearing small bowel in the pelvis with wall thickening (circled).

While in the emergency department, the first imaging performed was an abdominal computed tomography (CT) scan, which showed an abnormal loop of the small bowel in the pelvis that was mildly distended with wall thickening, raising concerns for possible ischemic bowel and SBO (Figure 1). The emergency department then consulted the surgery department, and it was decided to operate on the patient.

Preoperative diagnosis

The preoperative diagnosis was SBO.

Treatment

The patient underwent an exploratory laparotomy with small bowel resection and adhesiolysis. A massively dilated small bowel was observed intra-operatively, and the site of collapsed bowel could be identified after massive adhesiolysis between loops of the small bowel and the abdominal wall. An obvious site of obstruction was noted. There was a mass in the small bowel, as well as a large quantity of scar tissue that could not be lysed; this segment of the small bowel was then resected and the mesentery taken down. Peritoneal metastases were not observed, and other organs were noted to be intact with no evidence of prior surgery. The resected segment of the small bowel was sent for pathological analysis.

Postoperative diagnosis

The postoperative diagnosis was SBO.

Outcome/progress

After recovering from anesthesia, the patient was followed in an inpatient setting, which was notable for persistent symptoms. Pathological analysis of the resected small bowel from the exploratory laparotomy revealed grade 2 infiltrating adenocarcinoma developed on a tubulovillous adenoma of size 2.3 x 2.0 x 0.6 cm, infiltrating and focally penetrating the muscularis propria. The tumor cells in this specimen tested positive for CK7, CK20, and CDX2 on immunohistochemistry.

**Figure 2:**
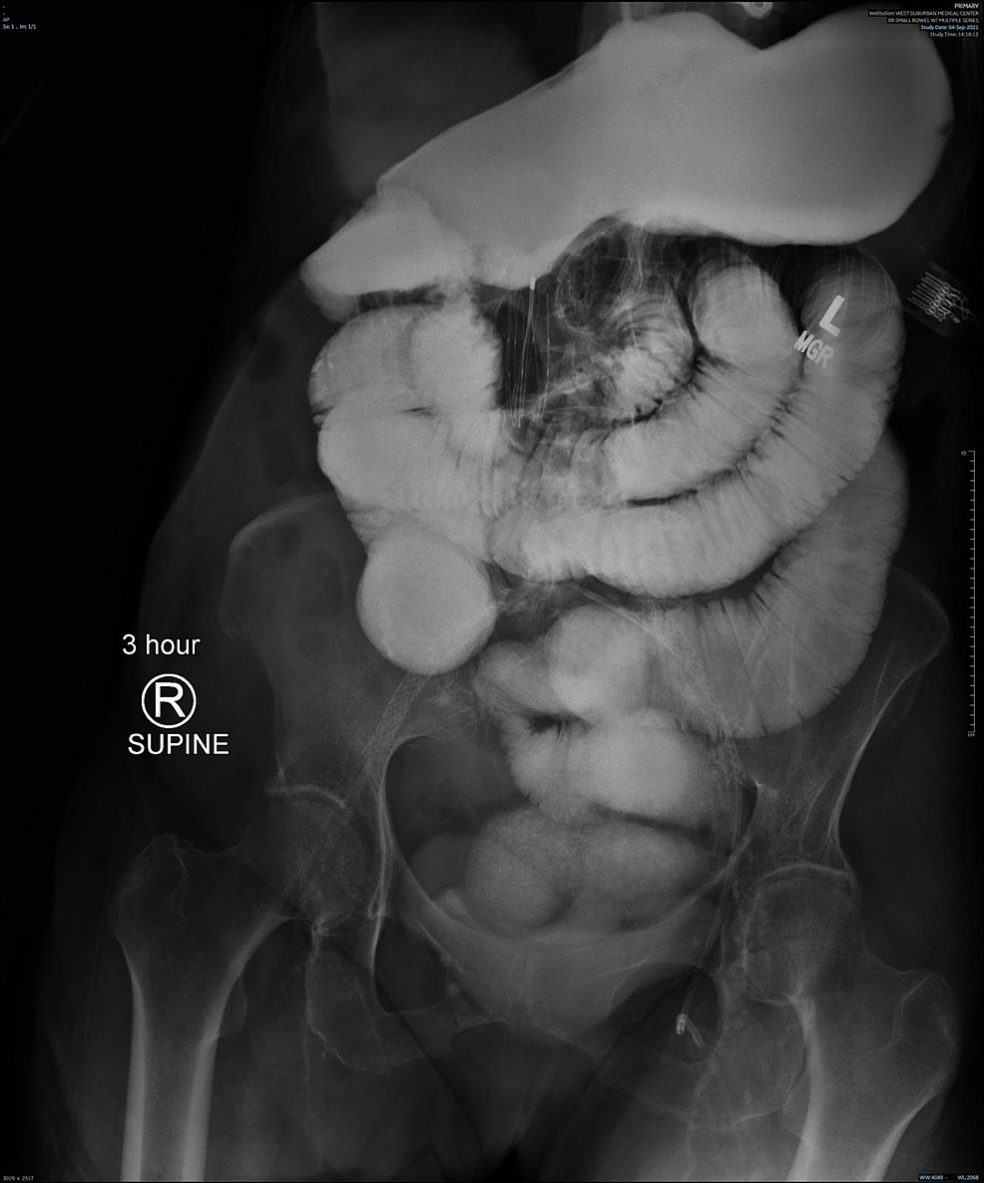
SBFT obtained three days after the first exploratory laparotomy, showing dilated small bowel at three hours, indicative of persistent SBO. SBFT, small bowel follow-through; SBO, small bowel obstruction

**Figure 3:**
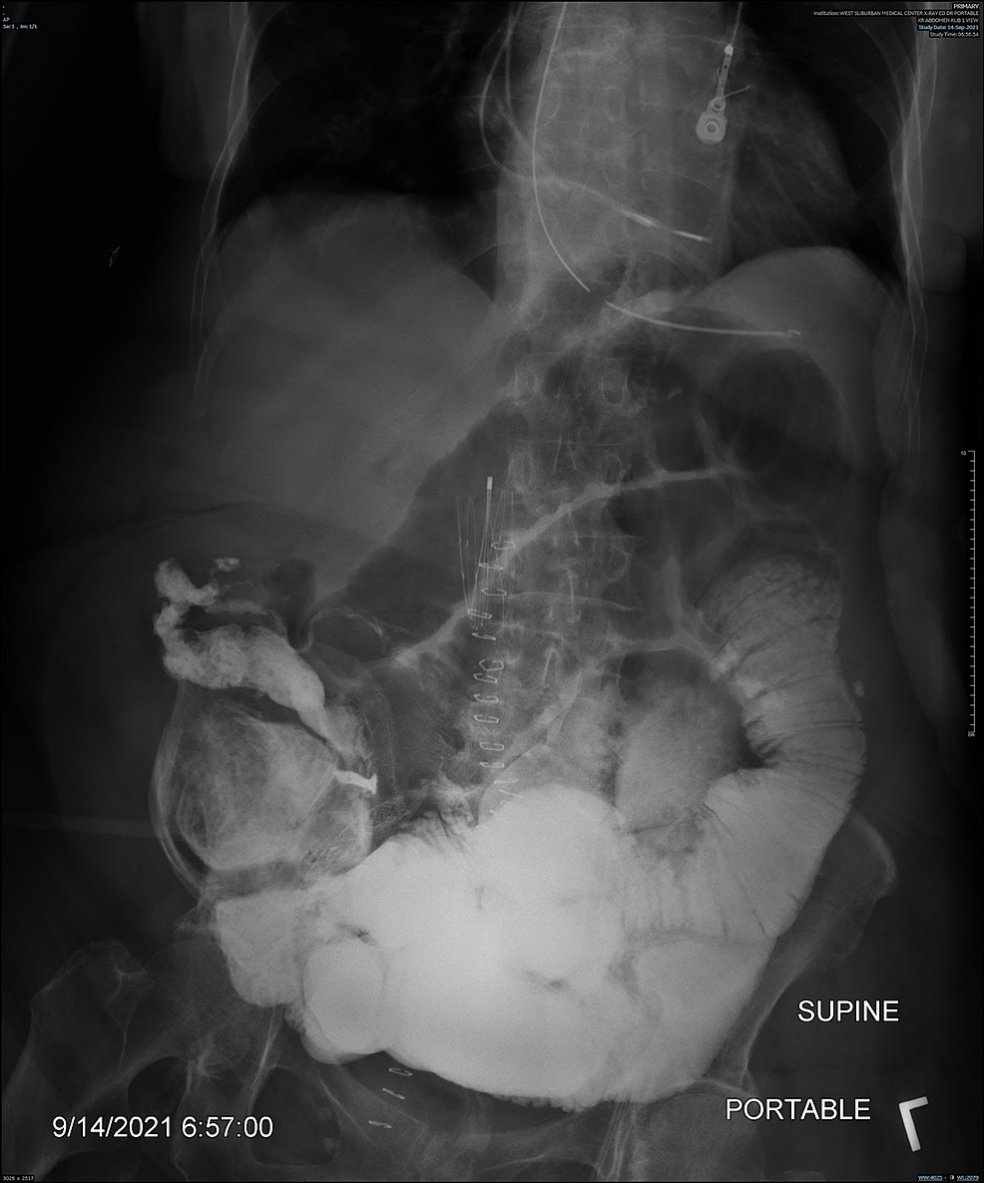
Abdominal radiograph (KUB) obtained 13 days after the first exploratory laparotomy, showing loops of the small bowel dilated by gas, fluid, and contrast, indicative of persistent SBO. KUB, kidney-ureter-bladder; SBO, small bowel obstruction

When persistent symptoms of obstruction were observed, a small bowel follow-through (SBFT) radiograph and abdominal radiograph (“kidney-ureter-bladder” or KUB) were obtained (Figures 2, 3); both forms of imaging suggested worsening SBO after the first exploratory laparotomy. The patient underwent a second exploratory laparotomy 13 days after the first exploratory laparotomy, this time with small bowel resection and an end ileostomy. Considerations such as the patient’s age, past medical history, and perceived ability to tolerate other medical procedures, including further surgery, played a part in the time between the patient’s first and second exploratory laparotomies.

After the second exploratory laparotomy, the patient was followed for 13 days in an inpatient setting, including management in the intensive care unit (ICU) and the skilled nursing facility. Pathological analysis of the resected small bowel from the second exploratory laparotomy showed serosal hemorrhagic adhesions with abscess formation, areas morphologically compatible with perforation, peri-intestinal adipose tissue with necrosis, acute hemorrhage, acute inflammation, and a single medium-sized arterial blood vessel with focal micro-calcifications, but no malignancy. The patient eventually had ample ostomy output indicative of a return of bowel function and was discharged home for outpatient follow-up with multiple services, notably oncology and gastroenterology.

## Discussion

SBO is predominantly caused by intestinal adhesions; most estimates indicate that 70-75% of all cases are attributable to adhesions [4,8]. Malignancy as a cause of SBO ranks a distant second, with estimates of SBO due to malignancy ranging from 5% [8] to 10%-20% [4]. Other causes of SBO include hernia (globally the most commonly attributed cause [1]), inflammatory bowel conditions, and radiation.

While a majority of patients treated (conservatively or operatively) for SBO do not have recurrence of SBO, nearly 20% are readmitted for recurrent SBO within five years of a prior treatment for SBO [9]. Operative intervention for adhesive SBO has been shown in recent reports to significantly reduce the recurrence of SBO compared to conservative/non-operative treatment [10], but these reports either do not differentiate between etiologies of SBO or focus specifically on adhesive SBO. We were unable to find reports in the literature discussing SBO recurrence/persistence rates for patients whose SBO etiology was malignancy.

Though details about our patient's prior history of cancer are unknown, given the lack of metastases and lack of evidence of prior surgeries (i.e., the presence of complete and intact organs) noted on imaging and her two operations, it is unlikely that our patient's malignancy is a recurrence of any prior cancer. These findings also suggest that our patient's small bowel adenocarcinoma was primary, which is uncommon [11]; as a cause of SBO, small bowel adenocarcinoma is a rare etiology [12].

The development of malignant/tumor tissue is known to be associated with inflammation, particularly in the gastrointestinal tract [13-15]. Inflammatory conditions also cause SBO, though less so than malignancy [1,2,4]. It is likely that the inflammation discovered in the resected small bowel specimen from our patient’s second exploratory laparotomy was the cause of the persistent obstruction in our patient. It is also possible that this inflammatory tissue was part of a process related to the expansion of malignant tissue discovered in the resected specimen from her first exploratory laparotomy, and that the two specimens together form a continuum of malignancy in her small bowel whose point of origin was in the first specimen. While frank malignancy is a known but uncommon cause of SBO, to our knowledge, ours is the first report of SBO that persisted after an initial small bowel resection due to small bowel adenocarcinoma and related processes.

The management of our patient was based on imaging findings, physical examination, and vigilant monitoring; in spite of her advanced age and past medical history, our patient was managed and treated in a timely manner, surviving two exploratory laparotomies in as many weeks, resulting in a successful outcome and restoration of bowel function.

## Conclusions

SBO due to malignancy may also be associated with inflammatory processes that can themselves cause obstruction. Therefore, patients with SBO of malignant tissue etiology should be monitored carefully after resection of malignant tissue because of the potential for malignancy-related processes such as inflammation to cause a recurrent/persistent obstruction. Relevant imaging studies include abdominal radiography (KUB) and SBFT with contrast, as these can help identify persistent SBO.
